# An Unusual Presentation of Costello Syndrome in a Boy with Precocious Puberty and Chiari I Malformation: A Case Report

**DOI:** 10.7759/cureus.78321

**Published:** 2025-02-01

**Authors:** Rita Lages Pereira, Ana Ribeiro, João Saraiva, JP Soares-Fernandes, Alexandra Gonçalves-Rocha, Ana Antunes, Maria Miguel Gomes, Sofia Martins

**Affiliations:** 1 Pediatrics Department, Unidade Local de Saúde de Braga, Braga, PRT; 2 Neuroradiology Department, Unidade Local de Saúde de Braga, Braga, PRT; 3 Medical Genetics Department, Unidade Local de Saúde de Braga, Braga, PRT; 4 Pediatric Endocrinology and Diabetology Unit, Pediatrics Department, Unidade Local de Saúde de Braga, Braga, PRT; 5 School of Medicine, University of Minho, Braga, PRT

**Keywords:** central precocious puberty, chiari type i malformation, costello syndrome, pediatrics endocrine, rare genetic diseases

## Abstract

Costello syndrome is a rare genetic disorder associated with developmental delay, short stature, and pubertal delay. However, a few cases of precocious puberty have been reported, reflecting the complex regulation of the hypothalamic-pituitary-gonadal axis affected by Harvey rat sarcoma viral oncogene homolog (HRAS) gene mutations. We present a case of a boy with Costello syndrome, heterozygous for a mutation in the HRAS gene, first seen in a pediatric endocrinology consultation at the age of nine years and seven months with central precocious puberty and short stature (-0.61 SD). The growth rate had accelerated from age seven years and six months. Pubarche and testicular enlargement began at age eight, and by nine years and seven months, the patient had reached Tanner stage V, with a testicular volume of 20 ml. Bone age was estimated to be 13 years. The brain magnetic resonance imaging (MRI) identified a Chiari type I malformation. At nine years and eight months, triptorelin treatment was initiated, leading to a reduction of pubic hair, stabilization of testicular volume, and a stature of -1.64 SD at the age of 13 years. Despite known factors influencing puberty, the precise physiological mechanisms behind its initiation remain unclear. This case provides valuable insights for understanding the genotype-phenotype relationship in Costello syndrome.

## Introduction

Costello syndrome is a rare genetic disorder caused by mutations in the Harvey Rat Sarcoma Viral Oncogene Homolog (HRAS) gene, leading to abnormal activation of the rat sarcoma/mitogen-activated protein kinase (RAS/MAPK) signaling pathway, which is part of a pathway that helps control cell growth and division [1]. The phenotype of Costello syndrome includes distinctive facial features, failure to thrive, feeding difficulties in infancy, short stature, developmental delay, hair and skin alterations (such as sparse, curly hair and loose, soft skin), heart involvement (congenital heart defects/arrhythmia) and a predisposition to malignancy, particularly rhabdomyosarcoma, and neuroblastoma. Pubertal delay is a typical feature of Costello syndrome, but cases of precocious puberty have been rarely reported [1,2]. Additionally, Costello syndrome patients are at an increased risk for neurological abnormalities, including seizures, hypotonia, and Chiari I malformation [2].

## Case presentation

A nine-year-seven-month-old boy with a known diagnosis of Costello syndrome presented for evaluation of suspected precocious puberty. His medical history included multiple neonatal complications such as respiratory distress syndrome, hypoglycemia, hypotonia, and a history of neonatal jaundice. Additionally, he had global developmental delay, feeding difficulties since he was born, tight Achilles tendons, and pectus carinatum. Coarse facial features and curly hair were evocative of Costello syndrome, and genetic testing revealed a heterozygous pathogenic mutation in the HRAS gene (c.34G>A), confirming the diagnosis.

**Figure 1:**
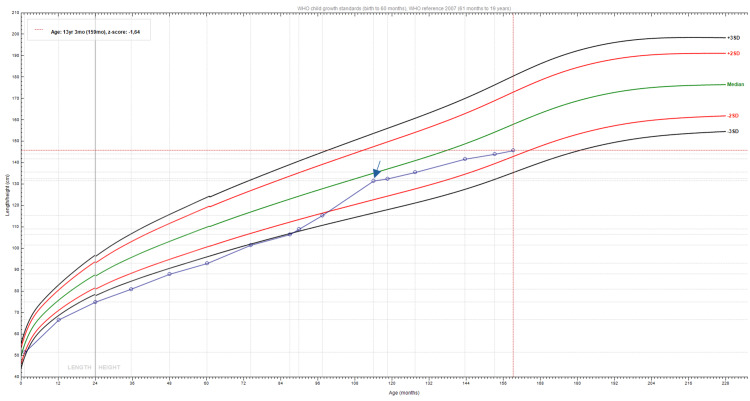
Growth curve of the patient The growth curve of the patient (blue line with points) shows a period of accelerated growth of around seven years and six months. Following the initiation of treatment with triptorelin (blue arrow), there is a noticeable stabilization in growth velocity.

The parents reported that at eight years of age, the patient began showing signs of early puberty, including pubic hair development and an increase in height velocity (Figure 1). On physical exam, he presented the typical facial features and height of 131.5 cm (-0.61 SD; family target height with +0.18 SD). Genital observation revealed Tanner stage V (testicular volumes of 20 ml). A bone age X-ray was interpreted as four years advanced of his chronological age. Laboratory testing confirmed central precocious puberty (CPP) with elevated luteinizing hormone (LH) at 5.60 mUI/mL (reference value (RV): 0.02-0.3 mUI/mL), follicle-stimulating hormone (FSH) at 4.45 mUI/mL (RV: 0.26-3.0 mUI/mL) and testosterone of 1163.90 ng/dL (RV: <2.5-10 ng/dL). No other dysfunction was noted (Table 1). Scrotal ultrasound revealed microlithiasis and a right epididymal cyst measuring 5 mm. Tumor markers were negative; however, despite this, the patient was evaluated by pediatric oncology and remains under regular ultrasound monitoring due to concerns about the increased risk of malignancy associated with Costello syndrome. No evidence of tumor or suspicion of malignancy has been observed till now.

**Table 1:** Laboratory evaluation of endocrine hormone levels and bone age over time DHEA-S - dehydroepiandrosterone sulfate; FSH - follicle-stimulating hormone; LH - luteinizing hormone; IGF-1 - insulin-like growth factor 1; Y - years; M - months † Initiated treatment with triptorelin at the first evaluation § Discontinued treatment with triptorelin

Laboratory tests	9Y 8M †	10Y 7M	12Y	13Y §
IGF-1 (ng/mL)	130	235	156	
FSH (mUI/mL)	4.45	0.9	0.63	0.44
LH (mUI/mL)	5.6	1.2	1.08	1
Testosterone (ng/dL)	1163.9	114.26	137.43	49.26
DHEA-S (ug/dL)			182	130
4-androstenedione (ng/mL)			0.75	0.84
17-hydroxyprogesterone (ng/mL)			0.6	0.76
Bone age	13Y 6M	14Y	14Y	14Y

Treatment with a gonadotropin-releasing hormone (GnRH) analog (triptorelin) was initiated at nine years and eight months, right after the first endocrinology appointment, to manage his pubertal development, with a dose of 11.25 mg administered intramuscularly every three months. Over the following years, the patient's growth (Figure 1) and puberty were closely monitored. His development showed stabilization, with no further signs of pubertal progression, stabilization of testicular volume, and reduction of hairiness, with a growth rate of 4 cm per year. Regular follow-up evaluations included ultrasound monitoring for the risk of malignancies associated with Costello syndrome, and no signs of tumors were noted. Laboratory values were consistent with a suppressed FSH, LH, and testosterone (Table 1). 

**Figure 2:**
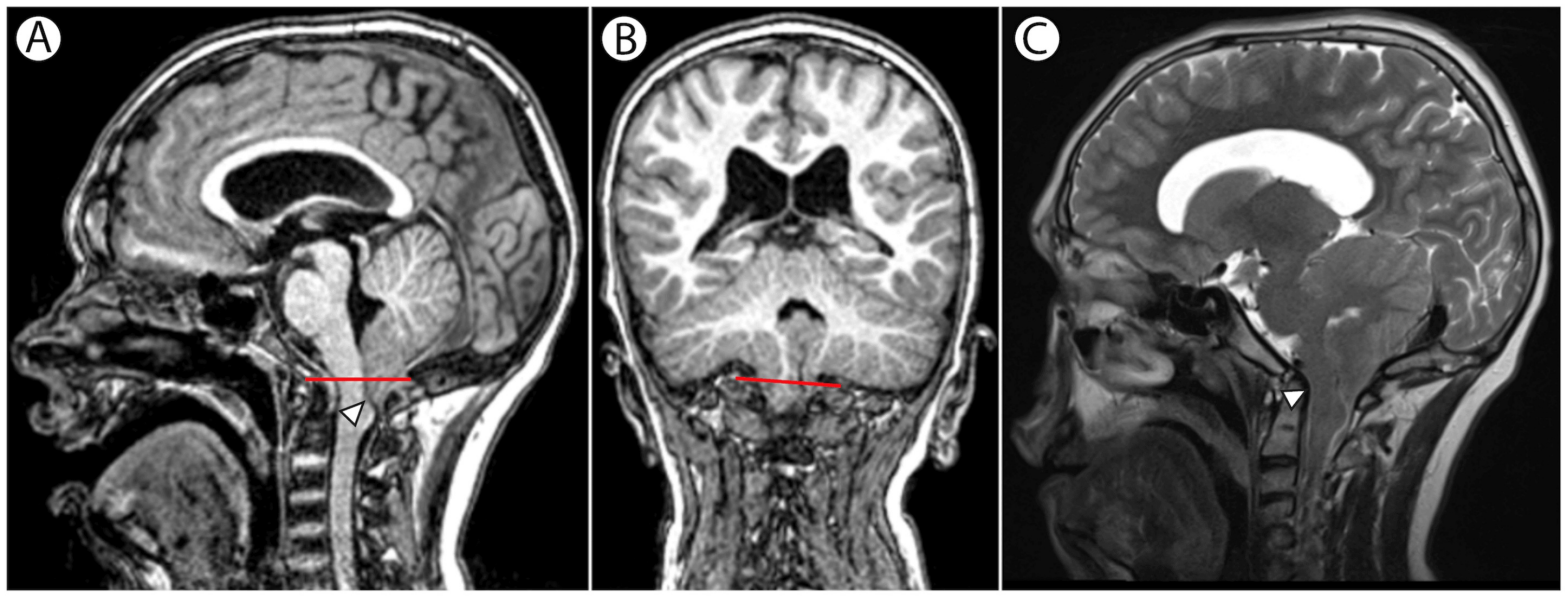
MRI of the brain A: 3D T1-MPRAGE sagittal reformat shows cerebellar tonsillar herniation below the foramen magnum, as indicated by McRae's line (red line); the posterior fossa appears hypoplastic, with caudal descent of the obex (arrowhead). B: 3D T1-MPRAGE coronal reformat highlights the extent of cerebellar tonsillar herniation (below the red line). C: Coronal T2-weighted image reveals CSF obliteration at the foramen magnum and ventral compression of the lower medulla (arrowhead), without edema or syringohydromyelia. T1-MPRAGE -  T1-weighted magnetization prepared rapid gradient echo; CSF - cerebrospinal fluid

An MRI was performed, and it revealed a Chiari I malformation with cerebellar tonsillar descent (Figure 2). The patient did not exhibit symptoms of hydrocephalus, such as headaches or neurological abnormalities. Evaluation by neurosurgery concluded that no surgical intervention was required, opting instead for close monitoring. A follow-up MRI at 13 years showed no progression of the Chiari I malformation, with no evidence of hydrocephalus or syringomyelia.

At 13 years of age, the decision was made to discontinue triptorelin treatment. At this age, his bone age was 14 years, with a height of 145.7 cm (-1.64 SD), bilateral testicular volume of 20 mL, pubic hair stage 2-3, and no axillary hair. The patient continues to be closely monitored for growth, pubertal progression, and associated complications of Costello syndrome.

## Discussion

This case highlights an unusual presentation of Costello syndrome with precocious puberty and Chiari I malformation. Although precocious puberty is not typically observed in Costello syndrome, some cases may occur [1,2]. CPP is defined as the early activation of the hypothalamic-pituitary-gonadal (HPG) axis, which manifests in boys by testicular enlargement before the age of nine years [3]. Advanced bone age, elevated gonadotropins, and pubertal testosterone levels were consistent with CPP in this case. The initiation of GnRH analog therapy managed the patient's pubertal progression, preventing more serious compromise in his adult height potential and psychosocial impact. The patient adhered to therapy with GnRH analogs for four years and three months, achieving stabilization of pubertal development and consistent growth patterns. The response to treatment in this case aligns with existing literature on the efficacy of GnRH analogs in managing CPP [3].

While most CPP cases are idiopathic, secondary causes, including intracranial pathology, exposure to high levels of sex steroids, or environmental factors, must be considered [4]. This patient's case raises the question of whether the Chiari I malformation and syrinx observed on brain imaging contributed to the premature activation of the HPG axis. Chiari malformations may disrupt normal hypothalamic-pituitary signaling. Structural brain anomalies like these can alter neuroendocrine regulation, potentially leading to early pubertal onset [5]. While such associations are documented in other neurological disorders linked to CPP [4], the precise mechanisms of Costello syndrome remain unclear. Further studies are needed to confirm this hypothesis and elucidate the pathophysiological mechanisms underlying CPP in patients with Costello syndrome and associated brain anomalies.

Another important concern in Costello syndrome is the increased risk of malignancy, which is a recognized feature of the condition [1,2]. Patients with Costello syndrome have a predisposition to certain tumors, most commonly rhabdomyosarcoma, neuroblastoma, and bladder carcinoma. In this case, the presence of scrotal microlithiasis and an epididymal cyst further underscores the need for vigilant monitoring, as such findings may raise concerns regarding potential malignancy. Regular imaging and laboratory evaluations are crucial in the ongoing surveillance of tumors in these patients, ensuring early detection and timely intervention if malignancy develops [6]. This adds another layer of complexity to the clinical management of this patient and highlights the importance of a proactive, multidisciplinary approach.

The findings in this case underscore the need for a comprehensive evaluation of neurological and endocrine features in Costello syndrome patients presenting with early or atypical pubertal signs. Neuroimaging should be a key part of the diagnostic workup in these cases, as structural brain abnormalities like Chiari I malformation may have significant clinical implications.

## Conclusions

In conclusion, this case broadens the clinical spectrum of Costello syndrome by documenting the occurrence of CPP in association with Chiari I malformation. It emphasizes the importance of a multidisciplinary approach, integrating endocrinology, genetics, and neurosurgery, to optimize the diagnosis, management, and long-term outcomes for these patients. Further research is essential to better understand the underlying mechanisms and refine management strategies for endocrine manifestations in Costello syndrome.
